# Persistence of SARS-CoV-2 infection on personal protective equipment (PPE)

**DOI:** 10.1186/s12879-021-06861-7

**Published:** 2021-11-19

**Authors:** Elizabeth Córdoba-Lanús, Omar García-Pérez, Sara Cazorla-Rivero, Francisco Rodríguez-Esparragón, José-Enrique Piñero, Bernardino Clavo, Jacob Lorenzo-Morales

**Affiliations:** 1 Instituto Universitario de Enfermedades Tropicales y Salud Pública de Canarias de La Universidad de La Laguna, La Laguna, Tenerife Spain; 2 Departamento de Medicina Interna, Dermatología y Psiquiatría, Universidad de La Laguna, La Laguna, Tenerife Spain; 3 Red Cooperativa de Enfermedades Tropicales (RICET), Instituto de Salud Carlos III, Madrid, Spain; 4 CIBER de Enfermedades Infecciosas, Instituto de Salud Carlos III, 28029 Madrid, Spain; 5 Research Unit, Hospital Universitario Dr. Negrín, Las Palmas de Gran Canaria, Spain; 6 Fundación Canaria del Instituto de Investigación Sanitaria de Canarias (FIISC), Las Palmas de Gran Canaria, Spain; 7 Departamento de Obstetricia, Ginecología, Pediatría, Medicina Preventiva y Salud Pública, Toxicología, Medicina Legal y Forense y Parasitología, Universidad de La Laguna, La Laguna, Tenerife Spain; 8 Chronic Pain Unit, Hospital Universitario Dr. Negrín, Las Palmas de Gran Canaria, Spain; 9 Radiation Oncology Department, Hospital Universitario Dr. Negrín, Las Palmas de Gran Canaria, Spain; 10 RETIC de Investigación en Servicios de Salud en Enfermedades Crónicas (REDISSEC), Instituto de Salud Carlos III, Madrid, Spain; 11 Instituto Universitario de Investigaciones Biomédicas y Sanitarias (IUIBS), BioPharm Group, Universidad de Las Palmas de Gran Canaria, Las Palmas de Gran Canaria, Spain

**Keywords:** COVID-19, SARS-CoV-2, RNA, PPE, Personal protective equipment, Face masks

## Abstract

**Background:**

SARS-CoV-2 stability and infection persistence has been studied on different surfaces, but scarce data exist related to personal protective equipment (PPE), moreover using realist viral loads for infection. Due to the importance for adequate PPE management to avoid risk of virus infection, RNA stability was evaluated on PPE.

**Methods:**

Persistence of SARS-CoV-2 infection and detection of genomic RNA in PPE (gowns and face masks) were determined by in-vitro assays and RT-qPCR, respectively. Samples were infected with a clinical sample positive for SARS-CoV-2 (Clin-Inf), and with a heat-inactivated SARS-CoV-2 strain sample (Str-Inf) as a control.

**Results:**

PPE samples infected with Clin-Inf were positive for the 3 viral genes on gowns up to 5 days post-infection, whereas these overall genes were detected up to 30 days in the case of face masks. However, gowns and FFP2 masks samples contaminated with Clin-Inf showed a cytopathic effect over VERO cells up to 5–7 days post-infection.

**Conclusions:**

SARS-CoV-2 RNA was detected on different PPE materials for 5 to 30 days, but PPE contaminated with the virus was infectious up to 5–7 days. These findings demonstrate the need to improve PPE management and to formulate strategies to introduce viricidal compounds in PPE fabrics.

## Background

The current pandemic caused by the novel human coronavirus named severe acute respiratory syndrome coronavirus 2 (SARS-CoV-2, previously known as HCoV-19 first) emerged in Wuhan, China, in 2019 [1].

Since then, several studies have been developed to assess the stability of SARS-CoV-2 on different surfaces to stablish the real risk of virus spread through fomites and airborne transmission (on surfaces or items). For instance, a previous study by van Doremalen et al. (2020), assessed the persistence of SARS-CoV-2 on different surfaces, and concluded that it was more stable on plastic and stainless steel than on copper and cardboard (up to 72 h for a 10^4^ viral titer initial infection) [2]. In the same way, others tested a similar human coronavirus, SARS-CoV-1 (P9) and HCoV (229E strains), reporting viral survival of 4–5 days at room temperature, on different surfaces such as aluminum, plastic, metal, wood, or paper using a viral load of 10^5^ [3, 4]. Furthermore, it has been observed that depending on the viral load, SARS-CoV-2 is able to survive for long periods (from 2 h to 9 days) [4, 5]. Moreover, the survival rate is also influenced by the environmental conditions; for example, temperatures higher than 30 ºC are known to significantly reduce the viral viability. However, the results from these studies were difficult to compare since viral loads and environmental conditions used in the infection assays were different in those studies. On the other hand, face masks from COVID-19 patients could carry viable SARS-CoV-2, specially on the inner surfaces [6]

The aim of the present study was to evaluate the persistence of viable SARS-CoV-2 and the detection of genomic viral RNA on different items related to personal protective equipment (PPE). This is a relevant issue considering the importance of the PPE management to reduce the risk of transmission to healthcare professionals and caregivers at health centers, especially under circumstances of low availability.

## Results

In PPE samples infected with heat-inactivated SARS-CoV-2 strain (Str-Inf), the amplification of viral genes was detected in face masks for 15 days and for 20 days in gowns samples. On the other hand, amplification for genes targeting the protein S and Orf1ab region persisted only for 5 days in case of gown samples but for 15 days for face masks (Table 1). The positive basal control of the RNA SARS-CoV-2 strain showed a median Ct value of 29 for the three genes.

**Table 1:** SARS-CoV-2 gene detection in PPE samples infected with a heat-inactivated SARS-CoV-2 strain 2019-nCoV/USA-WA1/2020 (Str-Inf)

PPE	Genes	5 days (Ct)^a^	10 days (Ct)	15 days (Ct)	20 days (Ct)
Gowns	N	29.4	31.5	32.8	33.8
	S	29.3	–	–	–
	Orf1ab	29.2	–	–	–
Face masks	N	30.7	32.8	33.7	–
	S	31.2	33.8	33.2	–
	Orf1ab	31.4	32.8	36.3	–

^a^Ct: Cycle threshold in relation to the number of cycles required for the fluorescent marked amplification to cross the threshold in the RT-qPCR reaction. Lower Ct values indicates higher viral load. Ct-values ≤ 37.0 were considered as positive

PPE samples infected with a clinical sample positive for SARS-CoV-2 (Clin-Inf) showed that detection of N protein, S protein and Orf genes was observed up to 5 days post-infection in gown samples, whereas these overall genes were detected for 30 days after infection in the case of face masks, showing a decreasing viral load during monitoring period (Table 2). The positive basal control of the clinical positive SARS-CoV-2 sample showed a median Ct value of 28.5 for the three genes.

**Table 2:** SARS-CoV-2 gene detection in PPE samples infected with SARS-CoV-2 positive human clinical sample (Clin-Inf)

PPE	Genes	5 days (Ct)	10 days (Ct)	15 days (Ct)	20 days (Ct)	25 days (Ct)	30 days (Ct)
Gowns	N	33.6	34.5	34.8	34.2	34.5	36.0
	S	31.8	–	–	–	–	–
	Orf1ab	33.3	–	–	–	–	–
Face masks	N	30.0	30.4	31.5	31.8	32.4	32.3
	S	29.6	30.8	31.0	32.1	32.3	32.0
	Orf1ab	29.7	30.2	32.3	32.5	33.2	33.3

^a^Ct: Cycle threshold in relation to the number of cycles required for the fluorescent marked amplification to cross the threshold in the RT-qPCR reaction. Lower Ct values indicates higher viral load. Ct-values ≤ 37.0 were considered as positive

**Fig. 1:**
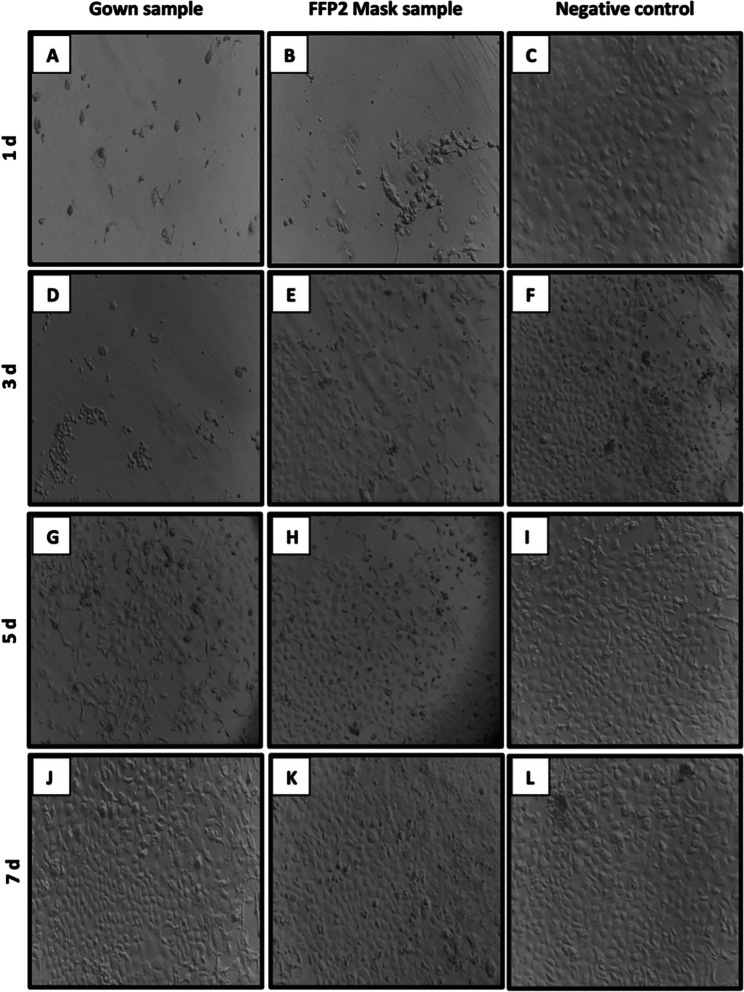
Images of VERO cells monolayer (× 20) after 24 h of incubation with virus infected PPE samples (1–7 days post-infection) and negative control of infection are shown. **A**, **D**, **G**, **J** show the decresent cytophatic effect observed in cells incubated with gown samples infected with SARS-CoV-2. **B**, **E**, **H**, **K** show the decreasing cytophatic effect observed in cells incubated with masks sample infected with SARS-CoV-2. **C**, **F**, **I**, **L** negative control consistent of VERO cells without infection. Images are representative of experiments performed in triplicate

In vitro experiments resulted in an immediate cytopathic effect of the VERO cells monolayer when infecting with PPE materials contaminated with SARS-CoV-2 Clin-Inf. An important decrease in the percentage of live cells after 24 h of incubation was observed for gown and FFP2 mask samples (Fig. 1) until the 7th day. VERO cells incubated in standard culture conditions were employed as a negative control of cytopathic effect and did not show alteration during the duration of the experiment.

## Discussion

In this study, the persistence of SARS-CoV-2 infection and the detection of genomic RNA of SARS-CoV-2 on PPE such as gowns and face masks were evaluated.

Our in vitro assays data support that PPE materials (gowns and masks) infected with a clinical sample positive for SARS-CoV-2 maintained its infectiveness up to 5–7 days post-infection. This is accordance to the 7 days previously described for surgical masks [7] and could have practical implications.

On the other hand, we detected viral genes in different types of PPE for up to 5 days after viral infection, and in some cases viral RNA was detected after 30 days (Table 2). Moreover, gown samples produced lower virus survival since the 3 viral genes were detected up to 5 days in both types of infection, with the genomic RNA from the heat-inactivated virus strain or with the clinical sample positive for SARS-CoV-2. In the case of face masks, the viral RNA stability was shown up to 15 days in the case of the control of genomic RNA viral strain and for 30 days when infected with the clinical sample positive for SARS-CoV-2.

SARS-CoV-2 has already been reported to persist on different surfaces for several days [4, 8, 9]. The limitation of most of these studies is that virus infections were performed with high viral loads resembling unrealistic scenarios [10]. In this study, we used a viral load corresponding to a more realistic situation, by using a human nasopharyngeal sample positive for SARS-CoV-2 to infect different PPEs samples.

Previously reported viral loads of SARS-CoV-2 in clinical samples ranged from a median of 7.99 × 10^4^ copies per mL in throat samples and 7.52 × 10^5^ in sputum samples post onset to overall higher values > 1 × 10^6^ copies per mL of the viral load early after onset [11]. Therefore, to accurately quantify the expected viral copy number of a clinical sample, a reliable and robust standard curve must be established. In our study, a nasopharyngeal sample was quantified by qPCR using a reliable standard curve performed with an appropriate reference material. From the resulting data, we decided to use a viral load concentration within approximate ranges (10^4^ viral copies) for infecting PPE samples trying to resemble a real situation.

The observed differences between the persistence of viral detection when infecting with a SARS-CoV-2 positive clinical sample (Clin-Inf) or viral RNA from a heat-inactivated SARS-CoV-2 strain (Str-Inf) used as a control, are consistent to their different nature. The virus contained in the nasopharyngeal cells presents its protective capsid which preserves its genomic material for a longer period, as it was observed in our study. Whereas the capsid of the heat inactivated commercial strain has functional and structural alterations that turn the virus non-infectious but more labile. Virus persistence was determined by the detection of viral RNA in different PPE materials.

Finally, we could observe that the composition material of face masks (FPP2 type), seems to be more suitable for virus stability probably due to its more porous nature, when compared to gown´s fabric [12]. Our results demonstrate that virus infectious viability on different surfaces should not only be tested by using molecular techniques, since genes could be detected longer than viral infective viability.

## Conclusions

PPE materials contaminated with SARS-CoV-2 remain infective between 5 to 7 days. Viral genomic RNA was detected in different PPE materials for 5 to 30 days in the case of face masks. We believe that these findings demonstrate the need of improving PPE fabric composition and to evaluate the addition of viricidal compounds to them. The responsible management of these PPE is crucial to avoid SARS-CoV-2 infection by contact of these items even many days after they are removed.

## Methods

In this study, we evaluated SARS-CoV-2 stability and viability in PPE gowns and FFP2 (KN 95) face masks infected by virus at room temperature. All experiments were carried out at the Biosafety Level 3 (BSL-3) facilities at the Instituto Universitario de Enfermedades Tropicales y Salud Pública de Canarias (La Laguna, Tenerife, Spain).

PPE gowns and face masks were infected with a human clinical nasopharyngeal sample positive for SARS-CoV-2 (Clin-Inf) and with genomic RNA from a heat-inactivated SARS-CoV-2 strain 2019-nCoV/USA-WA1/2020 (ATCC® VR-1986HK™) hereafter referred to as Str-Inf for simplicity, as a positive control.

Briefly, PPE gowns and masks samples were cut in pieces of around 20 × 10 mm. Each sample piece was infected with micro-droplets that accounted a total of 10 µL SARS-CoV-2 positive human nasopharyngeal sample with specific transportation medium (NEST Scientific) at a concentration of 10^3^ copies/µL, and with 10 µL of genomic RNA from SARS-CoV-2 strain (10^3^copies/µL) as control. PPE samples were stored at room temperature until infection of VERO cells and subsequent genetic analysis was performed.

At each evaluation time (between 2 to 30 days), two samples from each PPE materials: gowns and face masks were analyzed. Genomic analyses by real-time polymerase chain reaction (RT-qPCR) were performed for each sample collected in duplicate. The main outcome was to evaluate the in vitro infectiveness of SARS-CoV-2 and its persistence by the detection of 3 viral genes assessed by RT-qPCR.

### In vitro SARS-CoV-2 viral infection

Vero E6 cells (ATCC CRL-1586) cultures were maintained in DMEM (Dulbecco´s Modified Eagle Medium) supplemented with 10% foetal bovine serum (FBS) and 100U/mL of penicillin–streptomycin and cultured at 37ºC and 5% CO_2_ (Gibco, Gran Island, NY, USA) in 6 and 24 well plates (Nunc, ThermoFisher, Madrid, Spain) until an almost confluent monolayer was formed (10^6^ cells/ml). For infection, monolayers were washed twice with phosphate buffered saline (PBS) and inoculated with a SARS-CoV-2 infected PPE sample kept in non-supplemented DMEM. Non-infected control cultures (mock/negative control) were prepared using non-supplemented DMEM as inoculum. A positive control of infection was carried out using the Human coronavirus 229E ATCC ® VR-740 ™ strain (ATCC, LG Promochem, Barcelona, Spain). Cell monolayers were checked daily under a Leica DM6000 inverted light microscope for the presence of cytopathic effects (CPE) for up to 48 h post infection. All procedures were performed in a biosafety level 3 laboratory at our Institution as mentioned before. Cell lysate was collected from wells by gentle scrapping and pipetting, for further RT-qPCR assays. All the experiments were done in duplicate.

### Real-time polymerase chain reaction (RT-qPCR)

RT-qPCR was used to detect viral RNA according to Spanish guidelines for biosafety level-2 facilities.

RNA was extracted using the Maxwell 16S Viral RNA Mini Kit (Promega. Madrid. Spain) following the manufacturer’s recommendation. Briefly, each PPE infected sample was placed in 500 µL of inactivated medium (NEST Scientific). In the same way, 200–300 ul of the cell lysates infected with SARS-CoV-2 PPE sample were analyzed by this method. After this step, 200–300 µL of the sample (PPE samples in inactivated medium or cell lysates) was mixed with 300 µL of lysis buffer and 30 µL of proteinase K, vortex for 20 s to proceed with the RNA extraction procedure (manufacturer instructions). The resulting RNA was eluted in 50 µL and conserved at – 20 ºC until further use. For the SARS-CoV-2 genes amplification the TaqPathTM 1-Step RT-qPCR Master Mix and TaqPath™ COVID-19 CE-IVD RT-qPCR Kit (Applied Biosystems. Thermo Fisher Scientific, Madrid, Spain) were used in the RT-qPCR assays following the manufacturer’s instructions. The positive SARS-CoV-2 human sample used for infecting PPE samples, was quantified in a RT-qPCR assay in relation to a standard curve performed with the genomic RNA from SARS-CoV-2 strain 2019-nCoV/USA-WA1/2020 (ATCC® VR-1986HK™) at the following concentrations: 10^4^ to 16 viral copies and amplified by using the same RT-qPCR conditions described before.

This Multiplex Assay allows the qualitative detection and characterization of SARS-CoV-2 RNA. Briefly, the kit included three assays that target SARS-CoV-2 genes (Genes ORF1ab, N Protein, S Protein), a control of RNA extraction the MS2 Phage Control and a positive TaqPath™ COVID-19 Control. All the experiments were performed in duplicate in a QuantStudio5™ Real-Time PCR System (Applied Biosystems). A lower cycle threshold value in the RT-qPCR indicates a higher viral load. Positive results were considered when three genes had Ct values ≤ 37. If only one of the target genes had a Ct value ≤ 37 and the other > 37, it was interpreted as a single-gene positive.

## Data Availability

All data generated or analyzed during this study are included in this published article.
